# Novel risk stratification with time course assessment of in-hospital mortality in patients with acute heart failure

**DOI:** 10.1371/journal.pone.0187410

**Published:** 2017-11-02

**Authors:** Takeshi Yagyu, Masahiro Kumada, Tsutomu Nakagawa

**Affiliations:** Department of Cardiology, Toyonaka Municipal Hospital, Toyonaka, Osaka, Japan; Universita degli Studi di Napoli Federico II, ITALY

## Abstract

**Background:**

Patients with acute heart failure (AHF) show various clinical courses during hospitalization. We aimed to identify time course predictors of in-hospital mortality and to establish a sequentially assessable risk model.

**Methods and results:**

We enrolled 1,035 consecutive AHF patients into derivation (n = 597) and validation (n = 438) cohorts. For risk assessments at admission, we utilized Get With the Guidelines-Heart Failure (GWTG-HF) risk scores. We examined significant predictors of in-hospital mortality from 11 variables obtained during hospitalization and developed a risk stratification model using multiple logistic regression analysis. Across both cohorts, 86 patients (8.3%) died during hospitalization. Using backward stepwise selection, we identified five time-course predictors: catecholamine administration, minimum platelet concentration, maximum blood urea nitrogen, total bilirubin, and C-reactive protein levels; and established a time course risk score that could sequentially assess a patient's risk status. The addition of a time course risk score improved the discriminative ability of the GWTG-HF risk score (c-statistics in derivation and validation cohorts: 0.776 to 0.888 [p = 0.002] and 0.806 to 0.902 [p<0.001], respectively). A calibration plot revealed a good relationship between observed and predicted in-hospital mortalities in both cohorts (Hosmer-Lemeshow chi-square statistics: 6.049 [p = 0.642] and 5.993 [p = 0.648], respectively). In each group of initial low-intermediate risk (GWTG-HF risk score <47) and initial high risk (GWTG-HF risk score ≥47), in-hospital mortality was about 6- to 9-fold higher in the high time course risk score group than in the low-intermediate time course risk score group (initial low-intermediate risk group: 20.3% versus 2.2% [p<0.001], initial high risk group: 57.6% versus 8.5% [p<0.001]).

**Conclusions:**

A time course assessment related to in-hospital mortality during the hospitalization of AHF patients can clearly categorize a patient's on-going status, and may assist patients and clinicians in deciding treatment options.

## Introduction

Acute heart failure (AHF) is a common and occasionally life-threatening disease. In aging societies, the number of patients diagnosed with AHF has been growing, with AHF expected to become a serious public health problem and heavy social burden [1–3]. As health care providers will probably have to treat such patients with relatively reduced physical and human resources in future, a more efficient approach for treatment would be needed to maintain current medical standards. Under such conditions, a risk assessment—based practical strategy may be considered as having a key role in achieving such an approach [4].

Several models of risk stratification for in-hospital mortality of AHF patients have been described [4–9]. Most of these are well-validated and easy to use; however, their predictive abilities are not strong enough to be relied on in clinical settings, so that the estimation of a patient’s prognosis is still mainly based on a clinician’s subjective view [9]. One reason for their modest abilities in the prediction of in-hospital mortality may be that such risk stratification models are based solely on the initial assessments performed at admission. Patients with AHF exhibit various clinical courses in an in-hospital setting, with some gradually becoming severe during hospitalization, even if not critical at the point of admission. Although the in-hospital mortality risk of AHF patients would likely to change depending on their time course of disease, sometimes dramatically, few methods exist to sequentially assess an AHF patient’s condition after admission.

The purpose of this study was to identify in-hospital parameters associated with in-hospital mortality, and to establish a novel and sequentially evaluable predictive model by using time course information, in addition to initial information obtained at admission.

## Methods

### Study design and population

This retrospective, observational, and single-center study was conducted between January 2009 and December 2013, enrolling a total of 1,062 consecutive AHF patients admitted to our hospital. A clinical diagnosis of AHF was made based on clinical presentation, echocardiography assessment, and laboratory testing of natriuretic peptides, and was additionally confirmed by clinical records based on Framingham criteria for the diagnosis of heart failure [10]. We did not include patients with acute myocardial infarction or acute pulmonary thromboembolism in this study group, even if they showed signs or symptoms of AHF. The following AHF patients were excluded from this study: patients transferred to other hospitals for additive and urgent treatment, such as surgical intervention or heart transplantation; patients with infective endocarditis; patients with cardiopulmonary arrest on arrival; patients requiring non-pharmacological treatment for circulation support, such as intra-aortic balloon pumping and extracorporeal membrane oxygenation; and patients who were discharged or who died on the first day of admission.

In this study, because patient information was anonymized and de-identified prior to analysis, written informed consent was not obtained from each patient. However, we publicized the study by posting a summary of the protocol (with an easily understood description) on the website of Toyonaka Municipal Hospital; the notice clearly informed patients of their right to refuse enrollment. These procedures for informed consent and enrollment were in accordance with the detailed regulations regarding informed consent described in the guidelines, and this study, including the procedure for enrollment, has been approved by the institutional ethics board of Toyonaka Municipal Hospital (2016-01-04).

### Outcome and data collection

The primary outcome was defined as all-cause in-hospital mortality. For the assessment of the time course of disease after admission, we collected clinical data from electronic medical records, not only at admission points but also over entire periods during hospitalization. To establish a novel predictive model, we divided the overall data into derivation and validation cohorts according to their admission dates. The former consisted of patients who were admitted up until December 2011, and the latter consisted of those who were admitted in January 2012 and thereafter.

### Candidate parameters for in-hospital risk assessment

For an initial assessment at admission, we utilized an existing score model: Get With The Guidelines—Heart Failure (GWTG—HF) risk scoring model, which is a well validated, widely accepted, and user-friendly scoring system for the risk stratification of the in-hospital mortality of AHF (S1 Table) [8]. Here, we excluded the parameter of “Race” from this assessment because the population in this study consisted of a single race.

For a time course assessment after admission, we focused on the following parameters, which could dynamically represent the condition of patients’ organs and were considered as possible predictors: the presence of intravenous administration of any inotropic agent such as dopamine, dobutamine, norepinephrine or epinephrine, the presence of respiratory support by positive airway pressure with or without an invasive procedure, maximal values of total bilirubin, blood urea nitrogen, creatinine, C-reactive protein level, white blood cell concentration, and minimum values of albumin, sodium, and hemoglobin levels and platelet concentration during blood testing in all in-hospital periods, except for the day of discharge or death.

### Risk model establishment and statistical analysis

To derive a simple model in the derivation cohort, continuous variables obtained after admission were converted into binary values based on optimal cut-off points, which were defined by drawing receiver operating characteristic (ROC) curves and assigning round numbers close to Youden’s indexes [11].

From 12 candidate parameters, the GWTG—HF risk score at admission and the above 11 binary values obtained during hospitalization, we selected predictive variables for the best model by a stepwise selection with a *P* value of 0.20 for a backward elimination method, and assigned proper scores on the basis of weights of beta covariates calculated by multivariate logistic regression analysis in the derivation cohort. In this analysis, missing data were imputed by their median values.

For time course assessments, we defined total scores of assigned points in selected variables at each time point as a “time course risk score”. Finally, we established a summed score of the initial risk score (GWTG—HF risk score) and the maximal time course risk score as a “total in-hospital risk score”.

In order to validate the model performance of the total in-hospital risk score, we drew ROC curves and compared values of c-statistics in this score model and in the GWTG—HF risk score model by methodology previously described by DeLong et al. [12]. Calibration of the novel model was also assessed by a Hosmer—Lemeshow test and visual plotting [13]. Performances in particular subgroups were also examined in an overall cohort.

Continuous variables were expressed as means ± standard deviations and compared by a Student’s *t* test for normal distribution, or expressed as medians with interquartile ranges and compared by a Mann—Whitney *U* test for skewed distributions. Categorical variables were reported as numbers and percentages, and compared by a chi-square test or Fisher’s exact test as appropriate. For all tests, a *p* value < 0.05 was considered statistically significant.

Data were analyzed with R software packages Version 3.0.2 and JMP software Version 8.0.2 (SAS Institute, Inc., Cary, NC, USA).

## Results

### Study population

Of 1,062 AHF patients, 27 were excluded according to the above-mentioned criteria, with a final total of 1,035 patients enrolled. Of these, 597 patients who were admitted during or before December 2011 constituted the derivation cohort, and 438 patients who were admitted during or after January 2012 constituted the validation cohort. Overall characteristics of patients and each cohort are shown in Table 1. For the overall cohort, the mean age was 77.5 years, the proportion of males was 52.4%, and the mean value of the left ventricular ejection fraction was 42.0%. With regard to the etiology of heart failure, 21.2% of all AHF patients exhibited ischemic heart disease.

**Table 1:** Baseline characteristics at admission.

	Total Cohort (n = 1035)	Derivation Cohort (n = 597)	Validation Cohort (n = 438)
Age (years)	77.5 ± 12.5	76.5 ± 12.8	78.8 ± 12.2
Male (n)	542 (52.4)	284 (47.6)	258 (58.9)
BMI (kg/m^2^)	22.4 ± 4.3	22.4 ± 4.1	22.4 ± 4.5
NYHA Classification			
IV	613 (59.2)	347 (58.1)	266 (60.7)
III	407 (39.3)	238 (39.9)	169 (38.6)
II	15 (1.5)	12 (2.0)	3 (0.7)
Prior HF Admission (n)	420 (40.6)	232 (38.9)	188 (42.9)
Heart Disease (n)			
Ischemic Heart Disease	219 (21.2)	155 (26.0)	64 (14.6)
Cardiomyopathy	276 (26.7)	151 (25.3)	125 (28.5)
Hypertensive Heart Disease	337 (32.6)	185 (31.0)	152 (34.7)
Valve disease	138 (13.3)	70 (11.7)	68 (15.5)
Right Heart Failure	27 (2.6)	13 (2.2)	14 (3.2)
Others	38 (3.7)	23 (3.9)	15 (3.4)
LVEF < 40% (n)	472 (45.7)	277 (46.5)	195 (44.7)
Comorbidities (n)			
HT	669 (64.6)	376 (63.0)	293 (66.9)
DM	314 (30.3)	189 (31.7)	125 (28.5)
DL	263 (25.4)	150 (25.1)	113 (25.8)
CAD	308 (29.8)	202 (33.8)	106 (24.2)
CVD	174 (16.8)	95 (15.9)	79 (18.0)
PAD	58 (5.6)	35 (5.9)	23 (5.3)
COPD	29 (2.8)	14 (2.3)	15 (3.4)
HD	9 (0.9)	6 (1.0)	3 (0.7)
Device Implantation			
PM	51 (4.9)	27 (4.5)	24 (5.5)
ICD and/or CRT	9 (0.9)	3 (0.5)	6 (1.4)
Prior Cardiac Surgery			
CABG	56 (5.4)	38 (6.4)	18 (4.1)
Valve disease	31 (3.0)	21 (3.5)	10 (2.3)
Others	11 (1.1)	10 (1.7)	1 (0.2)
Malignancy			
Past	123 (11.9)	66 (11.1)	57 (13.0)
Current	33 (3.2)	20 (3.4)	13 (3.0)
Examination			
Systolic BP (mmHg)	146.8 ± 33.5	148.5 ± 33.6	144.4 ± 33.2
Diastolic BP (mmHg)	84.4 ± 23.3	85.4 ± 23.2	83.1 ± 23.4
Heart rate (bpm)	93.4 ± 27.6	94.6 ± 27.1	91.8 ± 28.1
Laboratory values			
Albumin (g/dL)	3.22 ± 0.48	3.17 ± 0.50	3.30 ± 0.44
Hemoglobin (g/dL)	11.8 ± 2.3	11.7 ± 2.3	12.0 ± 2.3
Platelets (×10^4^/μL)	19.0 [14.8–23.6]	19.1 [15.1–23.7]	19.0 [14.4–23.6]
WBC (×10^2^/μL)	69.0 [54.0–88.0]	70.0 [56.0–89.0]	66.5 [52.0–87.0]
Total Bilirubin (mg/dL)	0.81 [0.58–1.15]	0.81 [0.58–1.22]	0.80 [0.56–1.11]
BUN (mg/dL)	25 [18–35]	25 [17.5–35]	25 [18–35]
Creatinine (mg/dL)	1.08 [0.80–1.50]	1.10 [0.80–1.53]	1.06 [0.82–1.47]
Sodium (mEq/L)	140 [137–142]	141 [138–143]	139 [136–141]
CRP (mg/dL)	0.50 [0.14–1.75]	0.59 [0.14–1.82]	0.39 [0.15–1.61]
BNP (pg/mL)	694 [329–1323]	705 [323–1405]	669 [335–1205]
Electrocardiogram			
Sinus rhythm (n)	578 (55.8)	345 (57.8)	233 (53.2)
Atrial fibrillation (n)	362 (35.0)	199 (33.3)	163 (37.2)
Pacing (n)	45 (4.3)	22 (3.7)	23 (5.3)
Others (n)	50 (4.9)	31 (5.2)	19 (4.3)
Echocardiography findings			
LVDd (mm)	52.1 ± 9.5	51.9 ± 9.7	52.4 ± 9.3
LVEF (%)	42.0 ± 16.7	42.0 ± 16.4	41.9 ± 17.1

Numeric values are expressed as n (%), mean ± standard deviation or median (interquartile range 25–75%).

ACE: angiotensin-converting enzyme; ARB: angiotensin II receptor antagonist; BMI: body mass index; BNP: brain natriuretic peptide; BP: blood pressure; BUN: blood urea nitrogen; CABG: coronary artery bypass grafting; CAD: coronary artery disease; COPD: chronic obstructive pulmonary disease; CRP: C-reactive protein; CRT: cardiac resynchronization therapy; CVD: cerebral vascular disease; DL: dyslipidemia; DM: diabetes mellitus; HD: hemodialysis; HF: heart failure; HT: hypertension; ICD: implantable cardioverter defibrillator; LVDd: left ventricular diastolic dimension; LVEF: left ventricular ejection fraction; NYHA: New York Heart Association; PAD: peripheral artery disease; PM: pacemaker; WBC: white blood cells

### Candidate variables for prediction of in-hospital mortality

Collected variables for risk assessment and in-hospital mortality are presented in Table 2. Of the total 1,035 patients, 86 (8.3%) died during hospitalization. Of all variables used to calculate a new risk model, missing values were as follows: 15 values of minimum albumin levels and five values of maximum total bilirubin levels. Baseline data used to calculate the GWTG—HF risk score did not have missing data.

**Table 2:** Initial risk assessment and patients’ data during hospitalization.

	Total Cohort (n = 1035)	Derivation Cohort (n = 597)	Validation Cohort (n = 438)
**Initial Risk Assessment**			
GWTH—HF risk score	37.7 ± 8.2	37.2 ± 8.1	38.4 ± 8.3
**In-hospital Treatment**			
Catecholamine Administration (n)	195 (18.8)	113 (18.9)	82 (18.7)
Respiratory Support (n)	158 (15.3)	88 (14.7)	70 (16.0)
**Laboratory Values During Hospitalization**			
Min Alb (g/dL)	2.71 ± 0.62	2.67 ± 0.66	2.77 ± 0.57
Min Hb (g/dL)	10.6 ± 2.3	10.5 ± 2.4	10.7 ± 2.3
Min Plt (×10^4^/μL)	15.7 [12.1–19.6]	15.9 [12.2–20.0]	15.6 [12.1–18.9]
Max WBC (×10^2^/μL)	81.0 [64.0–105.0]	82.0 [65.0–106.0]	80.0 [63.0–105.0]
Max T-Bil (mg/dL)	0.98 [0.73–1.43]	0.98 [0.72–1.47]	0.99 [0.75–1.39]
Max BUN (mg/dL)	36 [27–52]	39 [28–55]	35 [26–49]
Max Creatinine (mg/dL)	1.32 [1.00–2.04]	1.36 [1.00–2.13]	1.30 [0.99–1.97]
Min Sodium (mEq/L)	136 [132–138]	137 [133–139]	135 [132–137]
Max CRP (mg/dL)	2.60 [0.71–7.21]	2.51 [0.71–7.16]	2.67 [0.72–7.22]
**Outcomes**			
In-Hospital Mortality (n)	86 (8.3)	47 (7.8)	39 (8.9)
Hospital Length of Stay (day)	22 [15–33]	22 [15–35]	21 [15–32]
Number of Blood Samples during Hospitalization	7 [5–11]	7 [5–11]	8 [5–11]

Numeric values are expressed as n (%), mean ± standard deviation or median (interquartile range 25–75%).

Alb: Albumin; BUN: blood urea nitrogen; CRP: C-reactive protein; GWTG—HF: Get With The Guidelines—Heart Failure; Hb: Hemoglobin; Max: maximum; Min: minimum; Plt: Platelets; T-Bil: total bilirubin; WBC: white blood cells

Associations of each variable with mortality and the optimal cut-off point to discriminate in-hospital risk are shown in S2 Table. Results of logistic regression analysis using such variables are shown in Table 3. Although individual variables showed a significant relationship to mortality, of 12 candidate parameters, five variables acquired after admission, other than the GWTG—HF risk score at admission, were picked out by stepwise backward selection: the presence of catecholamine administration, the minimum platelet concentration, and maximum blood urea nitrogen, total bilirubin, and C-reactive protein levels. Based on the relative proportion of each beta coefficient value to that of the GWTG—HF risk score, a risk scoring model derived from data obtained during hospitalization, termed a time course risk score, was constructed (Table 4). The formula for risk estimation calculated by logistic regression analysis for the derivation cohort was as follows:

$$\begin{array}{l} \text{Log}\;\text{odds}\;\text{of}\;\text{in-hospital}\;\text{mortality}\;=\;\\ 0.1078\;\times \;[\text{Total}\;\text{in-hospital}\;\text{risk}\;\text{score}]\;-\;9.0761 \end{array}$$

$$\begin{array}{l} {}[\text{Total in-hospital risk score}]\;=\\ {}[\text{GWTG-HF risk score (with the exception of racial score)}]\;\\ +\;[\text{maximal time course risk score during hospitalization}] \end{array}$$

**Table 3:** Logistic regression analysis on in-hospital mortality in derivation cohort.

	Univariate		Multivariate		
	OR [95% CI]	*P* value	β-coefficient	OR [95% CI]	*P* value
**Predictive Score Model at Initial Assessment**					
GWTG—HF risk score (per 10 points increase)	1.15 [1.11–1.21]	<0.0001	0.912	2.49 [1.56–4.10]	<0.0001
**Binary Variables in Time Course Assessment**					
Catecholamine Administration	8.06 [4.34–15.30]	<0.0001	0.945	2.58 [1.20–5.54]	0.015
Respiratory Support	3.43 [1.75–6.50]	0.0005			
Min Alb < 2.5 g/dL	3.41 [1.87–6.33]	<0.0001			
Min Hb < 10.0 g/dL	3.48 [1.84–6.97]	<0.0001			
Min Plt < 15.0×10^4^/μL	5.55 [2.81–12.00]	<0.0001	0.971	2.64 [1.20–6.18]	0.015
Max WBC ≥ 10000/μL	2.39 [1.31–4.38]	0.0047			
Max T-Bil ≥ 1.2 mg/dL	3.13 [1.71–5.87]	0.0002	0.581	1.79 [0.85–3.79]	0.126
Max BUN 2265 60 mg/dL	11.05 [5.84–21.79]	<0.0001	1.688	5.41 [2.62–11.45]	<0.0001
Max Creatinine ≥ 2.0 mg/dL	4.27 [2.33–8.00]	<0.0001			
Min Sodium < 135 mEq/L	2.79 [1.53–5.15]	0.0009			
Max CRP ≥ 3.0 mg/dL	3.43 [1.81–6.87]	0.0001	0.517	1.68 [0.78–3.72]	0.188

Six variables in multivariate analysis were adopted on the basis of the backward stepwise selection.

Alb: Albumin; BUN: blood urea nitrogen; CI: Confidence Interval; CRP: C-reactive protein; GWTG—HF: Get With The Guidelines—Heart Failure; Hb: Hemoglobin; Max: maximum; Min: minimum; OR: Odds Ratio; Plt: Platelets; T-Bil: total bilirubin; WBC: white blood cells.

**Table 4:** Time course risk scores.

Variables	Score Point
BUN ≥ 60 mg/dL	20
Catecholamine Administration	10
Platelets < 15.0×10^4^/μL	10
Total Bilirubin ≥ 1.2 mg/dL	5
CRP ≥ 3.0 mg/dL	5

BUN: blood urea nitrogen; CRP: C-reactive protein.

**Fig 1:**
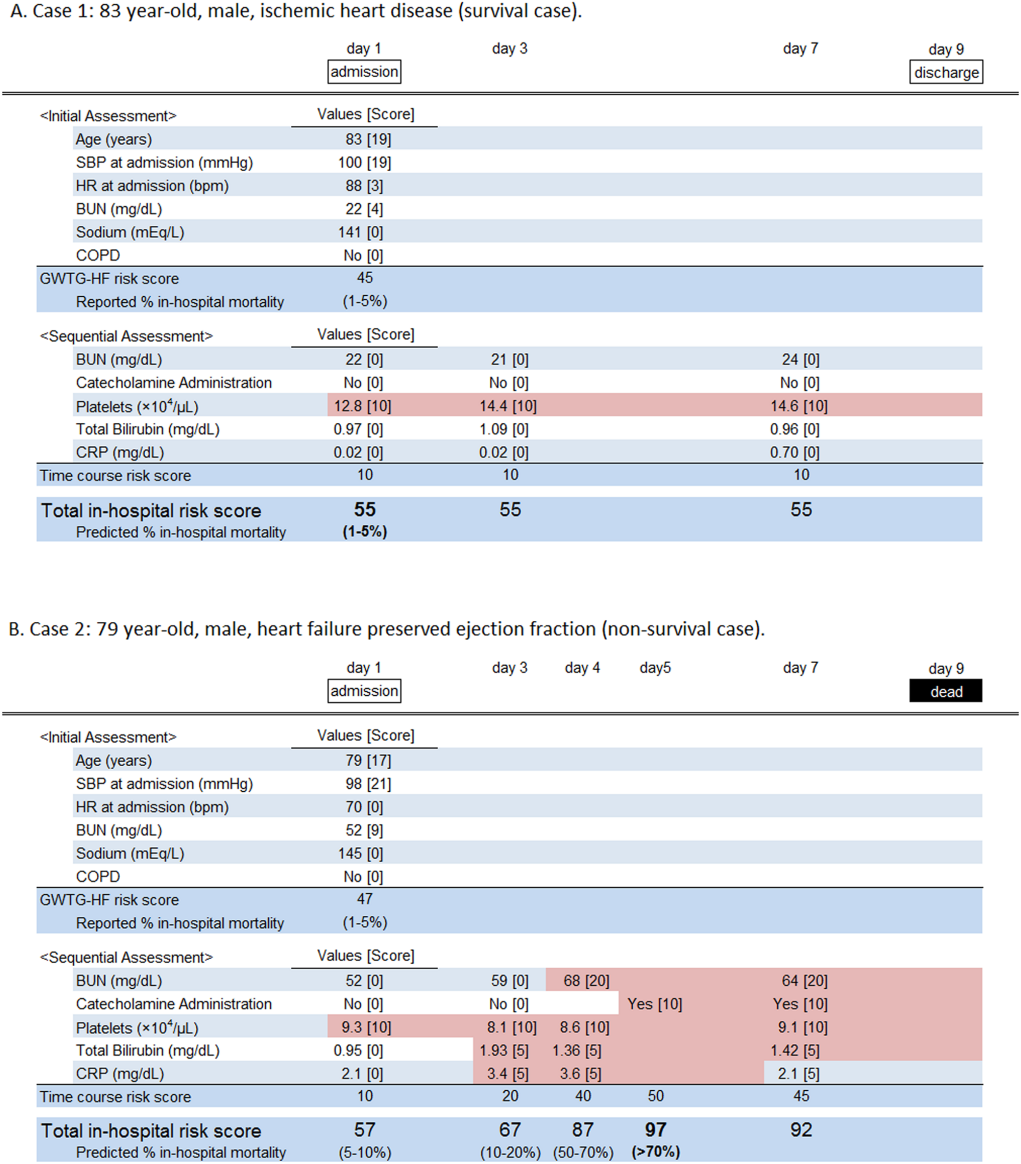
Case examples for time course risk scoring. (A) An 83-year-old male with ischemic heart disease was admitted for the exacerbation of heart failure. His Get With the Guidelines—Heart Failure (GWTG—HF) risk score, with the exception of a racial score, was 45 points at admission, which implied a 1 to 5% estimation of in-hospital mortality according to the original report [7]. In a sequential risk assessment over hospitalization, a maximal value of the time course risk score was 10 points on day one. Thus, the maximal value of the total score over the patient’s hospital stay was 55, which suggested 1 to 5% of in-hospital mortality according to our study. The patient’s condition improved rapidly and he was discharged on day nine. (B) A 79-year-old male with heart failure and a preserved ejection fraction had a GWTG—HF risk score, with the exception of a racial score, of 47 points at admission, which also implied a 1 to 5% estimation of in-hospital mortality. His time course risk score was also 10 points on day one; however, with his disease condition worsening, his time course risk score gradually increased to a maximal value of 50 points at day five, which implied an over 70% estimation of in-hospital mortality. He did not show any improvement in his condition and finally died on day nine. BUN: blood urea nitrogen; COPD: chronic obstructive pulmonary disease; CRP: C-reactive protein; HR: heart rate; SBP: systolic blood pressure.

Case examples of sequential assessments for in-hospital mortality using this scoring method are presented in Fig 1.

### Model validation

**Fig 2:**
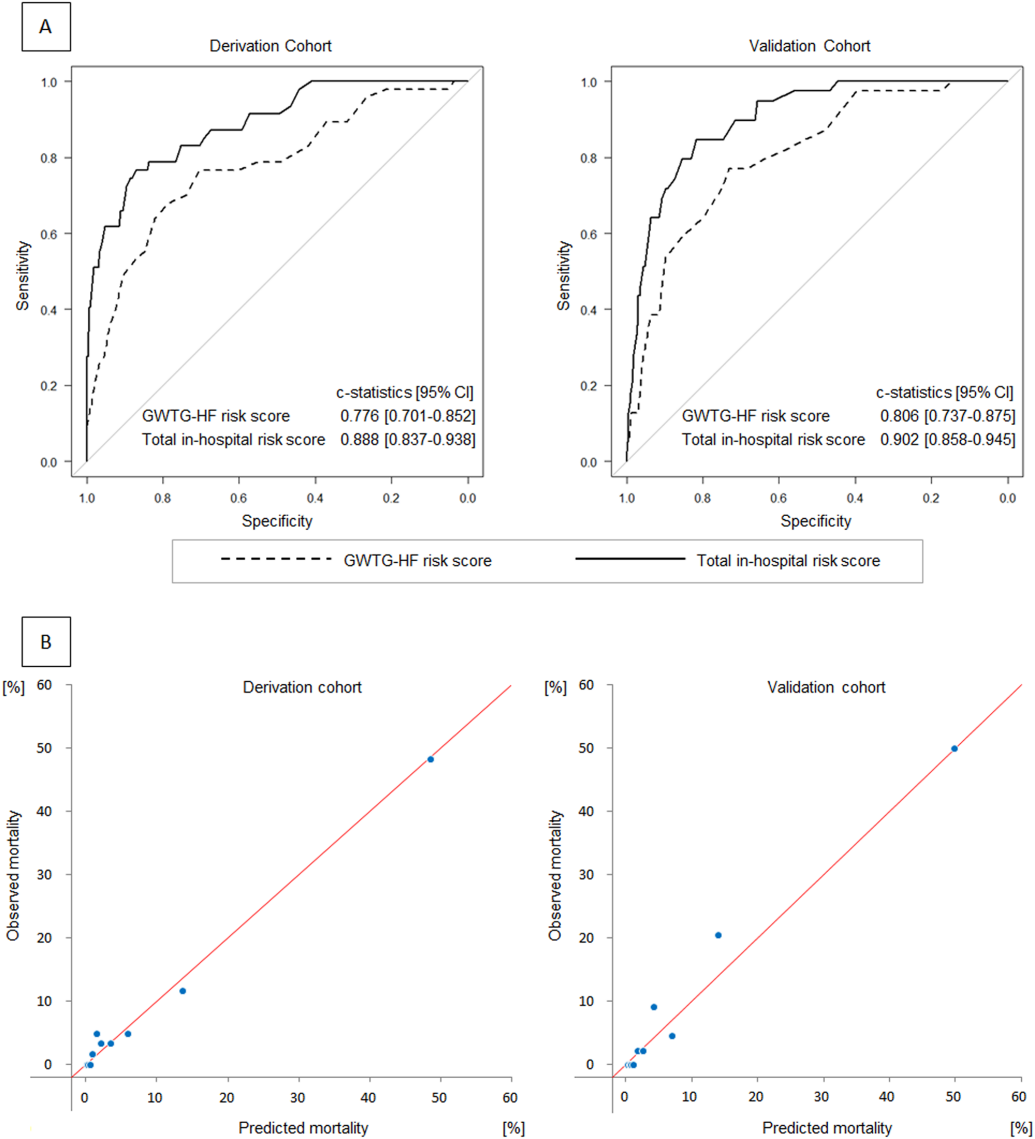
Discrimination and calibration of the novel risk model. (A) Receiver operating characteristic curves for the GWTG—HF risk score (broken line) and total in-hospital risk score (solid line) in derivation and validation cohorts. (B) Calibration plots of observed to predicted mortalities according to deciles in derivation and validation cohorts.

An addition of time course risk score to the GWTG—HF risk score significantly improved the c-statistics of the risk model for derivation and validation cohorts from 0.776 (95% confidence interval [CI]: 0.701–0.852) to 0.888 (95% CI: 0.837–0.938, *p* = 0.002), and from 0.806 (95% CI: 0.737–0.875) to 0.902 (95% CI: 0.858–0.945, *p* < 0.001), respectively (Fig 2A). The cut-off point for in-hospital mortality, which was defined on the basis of Youden’s index, in the derivation cohort was 64, with a sensitivity of 0.766 and a specificity of 0.871. For the validation cohort, the sensitivity of the same score point was 0.795 and its specificity was 0.855. For the calibration of this model, plots of predicted and observed in-hospital mortalities in each decile are shown in Fig 2B. Hosmer—Lemeshow chi-square statistics for the two cohorts were 6.049 (*p* = 0.642) and 5.993 (*p* = 0.648), respectively.

The c-statistics of subgroups for all study patients are shown in S3 Table. A significant improvement in discriminative ability, compared with the GWTG—HF risk score model, was observed for most of the subgroups.

**Fig 3:**
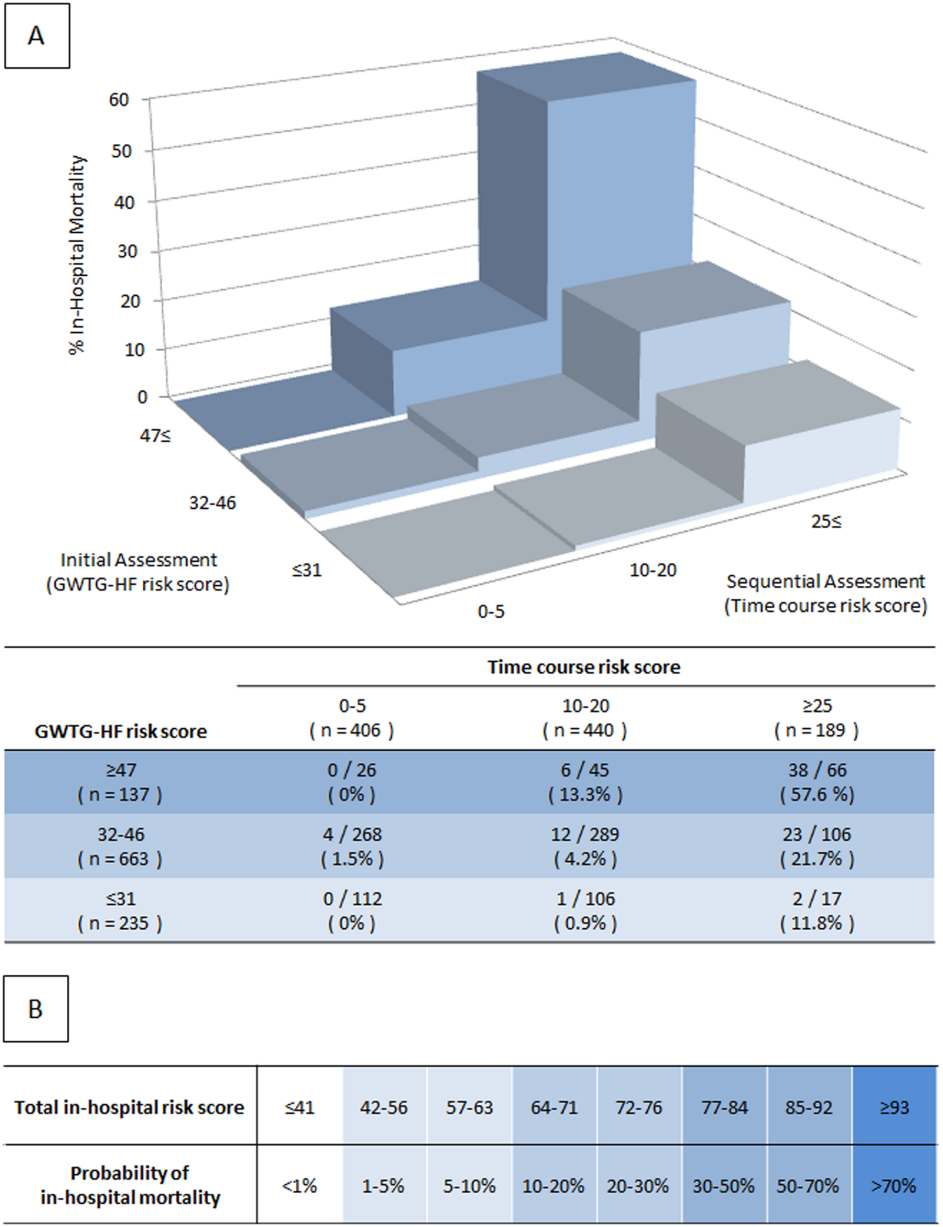
Risk distribution in the overall cohort. (A) Subdivision, in the overall cohort, of the GWTG—HF risk score by three classes of time course risk scores. (B) Risk chart of the total in-hospital risk score model.

Actual patients’ distributions of GWTG—HF, time course and total in-hospital risk scores for the overall cohort are shown in S1A–S1C Fig. We divided the overall cohort into three groups according to GWTG—HF (low: ≤ 31, intermediate: 32 to 46, high: ≥ 47) and time course risk scores (low: ≤ 5, intermediate: 10 to 20, high: ≥ 25). The in-hospital mortality rate for each risk status at admission and during hospitalization is presented in Fig 3A. In each risk group at admission, the in-hospital mortality rate in the high time course risk score group was higher than in that in the low and intermediate time course risk score groups (initial low risk group: 11.8% (2/17) versus 0.46% (1/218) [*p* = 0.014], initial intermediate risk group: 21.7% (23/106) versus 2.9% (16/557) [*p* < 0.001], initial high risk group: 57.6% (38/66) versus 8.5% (6/71) [*p* < 0.001]). A practical risk chart of the estimated in-hospital mortality from the above formula is presented in Fig 3B.

## Discussion

### Main findings

We found that a gradual progression of specific functional in-hospital disturbances, namely renal dysfunction, circulatory failure, coagulation disorders, liver dysfunction, and an inflammatory response, were strongly associated with the subsequent in-hospital deaths of AHF patients. Based on this finding, we proposed a novel in-hospital risk stratification method, which was established by adding information collected after admission to a traditional risk score model at admission. Time course assessments made during the hospitalization of patients with AHF may provide further information for in-hospital risk predictions. Specifically, such a scoring model is able to represent a serial “on-going” risk status.

### In-hospital mortality risk assessment

Risk stratification plays an important role in clinical practice for AHF patients, providing severe cases with adequate intensive treatment and preventing mild cases from receiving excessive, unnecessary care [14]. It may also help critical patients, their families, and health care providers make decisions on end-of-life care [14,15]. With regard to older patients with heart failure, a cohort that has been gradually growing in aging societies, appropriate end-of-life care should be provided but this is often not easy to carry out. Reasons for this may be that the time course of AHF during a hospital stay often varies and is also often unpredictable [16]. Regarding the examples of Fig 1, patient GWTG—HF risk scores and predicted in-hospital mortality at admission were similar; however, their clinical courses after admission differed greatly. This proposed model may allow patients and the people involved in their care to make the most appropriate medical choices under continually varying circumstances.

Several scoring models for the in-hospital mortality of patients with AHF have been described; these showed modest prognostic abilities and were quite similar (c-statistics: 0.70–0.80) [5–8], probably because their predictions were largely based on assessment at admission alone. Thus, at the point of admission, this level of predicting in-hospital mortality seems to be an upper limitation. Nonetheless, since AHF patients require on-going treatment after admission and their status may alter over time during hospitalization, a sequential assessment of each patient’s status should be made on a continual basis during in-hospital care. Although we were forced to assess each patient’s condition after admission, mainly in a subjective manner, using such a score model would allow us to make an objective and more precise assessment of the risk of in-hospital mortality, even after admission.

### Time course assessment

The risk prediction for individual AHF patients is likely to be challenging. One reason may be that patients’ conditions during acute phases may be too complex and fluctuant to be appropriately assessed [16]. Although several variables during the hospitalization of AHF patients are individually associated with in-hospital risk [17–19], to date, no method exists that can bundle such multiple risk factors during the evaluation of in-hospital mortality. To the best of our knowledge, this is the first report of a sequential and comprehensive assessment method of in-hospital mortality in AHF patients. This dynamic, in-hospital risk assessment could not only prompt health care providers to adjust their treatment strategies accordingly, but may also help patients and their families understand their loved one’s “on-going” condition and have the foresight to prepare for the worst [16,20].

The Sequential Organ Failure Assessment (SOFA) score is one of the few assessment methods available that can assess the severity of disease during hospital stays [21–23]. This scoring method was developed to quantify the severity of illness, not to predict mortality, and has been specifically used in critical care settings. It is unknown whether the SOFA score is efficient in predictions of in-hospital mortality in AHF patients. In comparison, our model was derived from a cohort of AHF patients, and produced in a manner that added in an already known risk assessment at the point of admission. It was also designed to be used easily. Thus, we considered that for AHF cases, our assessment method is more useful in the time course prediction of in-hospital mortality.

### Limitations

Our investigation had several limitations. First, this was a retrospective single-center study of a relatively small size. Although good discrimination and calibration were indicated in the validation cohort, it is uncertain whether the results of this study can be applied to other institutions or different patient populations, especially patients requiring intensive treatment, such as surgical interventions or heart transplantation. Furthermore, in Japan, the hospital length of stay of patients with AHF is generally longer than that in other countries [24]. In our study, the duration of hospital stay of our patients was of average length for Japan, but was much longer compared with other countries [25,26]. Whether our findings can be applied to patients with AHF in countries with a shorter hospital length of stay remains to be examined. Second, predictive parameters included the type of treatment, which involved pharmacological circulatory support. Although the necessity for inotropic agent administration would reflect the severe condition of AHF patients, whether the administration itself is harmful or should be avoided remains inconclusive. Finally, blood sampling was undertaken in each clinical setting, not at scheduled time points. In some cases, for example in the full recovery phase, or conversely during intense critical conditions in which recovery was unlikely, frequent laboratory tests were often withheld. Although this may have led to a misestimate of the predictive ability of the model, we consider this model as reliable enough to be applied to clinical settings according to the results of this validation study.

Regardless of the limitations outlined above, this scoring model provided sequential and more accurate predictions of in-hospital mortality than any previously reported models. Further studies with larger numbers of patients in various clinical settings are required to confirm these results.

## Conclusion

In addition to a traditional assessment at the time of admission, a comprehensive time course assessment during hospitalization may predict in-hospital mortality more accurately. The novel risk stratification model described herein may provide patients, their families and clinicians with valuable information in a hospital setting.

## Supporting information

S1 Fig Distribution of patient numbers and mortality rates by each risk score in the overall cohort. (A) GWTG—HF risk score with the exception of racial score. (B) Time course risk score. (C) Total in-hospital risk score (GWTG—HF risk score with the exception of racial score + Time course risk score).
(TIF)

S1 Table Get With The Guidelines Heart—Failure (GWTG—HF) risk model. BP: blood pressure; BUN: blood urea nitrogen; COPD; chronic obstructive pulmonary disease.
(DOCX)

S2 Table Candidate variables obtained during hospitalization in derivation cohort for in-hospital mortality risk assessment. Numeric values are expressed as n (%), mean ± standard deviation or median (interquartile range 25–75%). Cut-off values in continuous values were decided based on maximum Youden’s indexes. Alb: Albumin; BUN: blood urea nitrogen; CRP: C-reactive protein; Hb: Hemoglobin; Max: maximum; Min: minimum; Plt: Platelets; T-Bil: total bilirubin; WBC: white blood cells.
(DOCX)

S3 Table Subgroup analysis on discrimination ability. AF: Atrial Fibrillation; CI: Confidence Interval; DM: Diabetes Mellitus; GWTG—HF: Get With The Guidelines—Heart Failure; LVEF: Left Ventricular Ejection Fraction; SR: Sinus Rhythm.
(DOCX)
