# Supplementary figures and images for: Novel risk stratification with time course assessment of in-hospital mortality in patients with acute heart failure
Source: PLoS One. 2017 Nov 2;12(11):e0187410. doi: 10.1371/journal.pone.0187410 (PMC5667756; doi:10.1371/journal.pone.0187410)

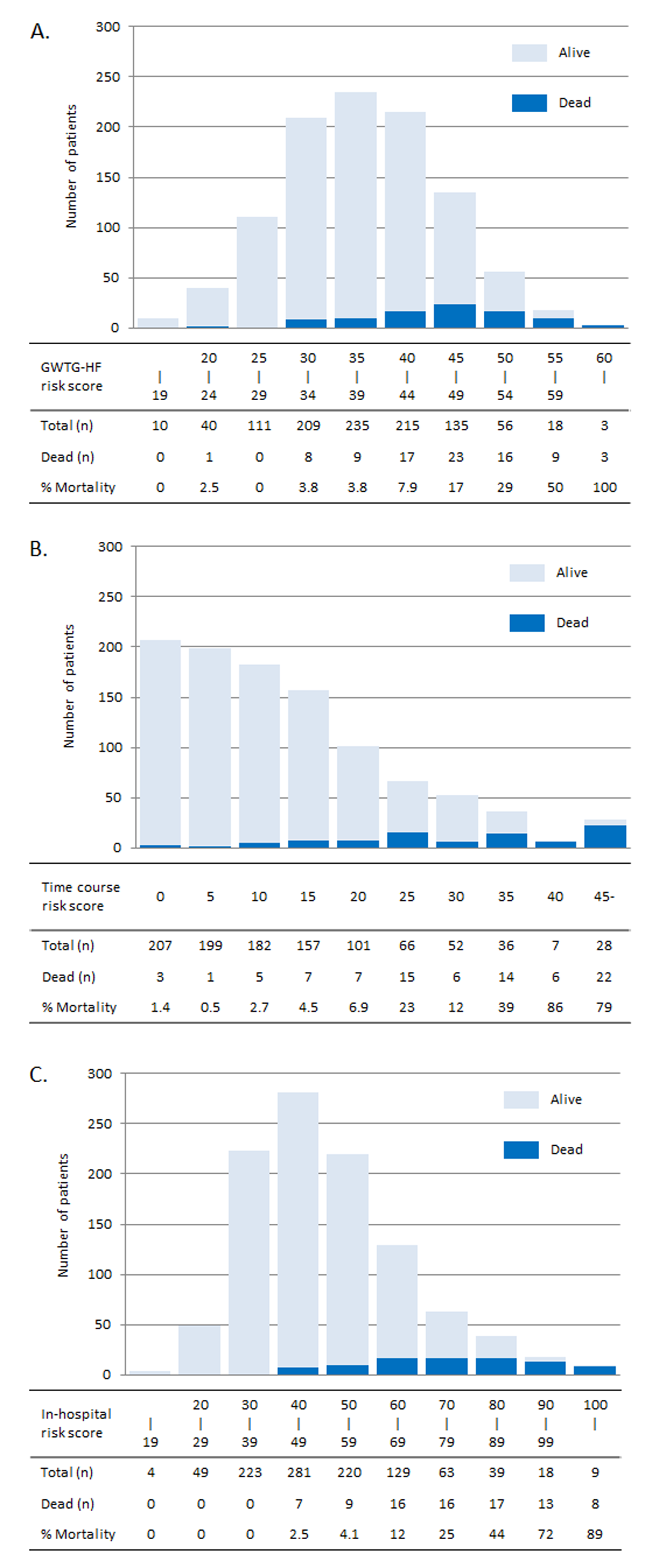

Supplement: S1 Fig — (A) GWTG—HF risk score with the exception of racial score. (B) Time course risk score. (C) Total in-hospital risk score (GWTG—HF risk score with the exception of racial score + Time course risk score). (TIF) [file pone.0187410.s001.tif]
