# Supplementary material for: Novel risk stratification with time course assessment of in-hospital mortality in patients with acute heart failure
Source: PLoS One. 2017 Nov 2;12(11):e0187410. doi: 10.1371/journal.pone.0187410 (PMC5667756; doi:10.1371/journal.pone.0187410)
Supplement: S1 Table — BP: blood pressure; BUN: blood urea nitrogen; COPD; chronic obstructive pulmonary disease. (DOCX) [file pone.0187410.s002.docx]

| Systolic BP  (mmHg) | Points | BUN  (mg/dL) | Points | Age  (years) | Points |
| --- | --- | --- | --- | --- | --- |
| 50-59 | 28 | ≤9 | 0 | ≤19 | 0 |
| 60-69 | 26 | 10-19 | 2 | 20-29 | 3 |
| 70-79 | 24 | 20-29 | 4 | 30-39 | 6 |
| 80-89 | 23 | 30-39 | 6 | 40-49 | 8 |
| 90-99 | 21 | 40-49 | 8 | 50-59 | 11 |
| 100-109 | 19 | 50-59 | 9 | 60-69 | 14 |
| 110-119 | 17 | 60-69 | 11 | 70-79 | 17 |
| 120-129 | 15 | 70-79 | 13 | 80-89 | 19 |
| 130-139 | 13 | 80-89 | 15 | 90-99 | 22 |
| 140-149 | 11 | 90-99 | 17 | 100-109 | 25 |
| 150-159 | 9 | 100-109 | 19 | ≥110 | 28 |
| 160-169 | 8 | 110-119 | 21 |  |  |
| 170-179 | 6 | 120-129 | 23 |  |  |
| 180-189 | 4 | 130-139 | 25 |  |  |
| 190-199 | 2 | 140-149 | 27 |  |  |
| ≥200 | 0 | ≥150 | 28 |  |  |
|  |  |  |  |  |  |
| Heart Rate  (bpm) | Points | Sodium  (mEq/L) | Points | Black Race | Points |
| ≤79 | 0 | ≤130 | 4 | Yes | 0 |
| 80-84 | 1 | 131-133 | 3 | No | 3 |
| 85-89 | 3 | 134-136 | 2 |  |  |
| 90-94 | 4 | 137-138 | 1 | COPD | Points |
| 95-99 | 5 | ≥139 | 0 | Yes | 2 |
| 100-104 | 6 |  |  | No | 0 |
| ≥105 | 8 |  |  |  |  |
