# Supplementary material for: Novel risk stratification with time course assessment of in-hospital mortality in patients with acute heart failure
Source: PLoS One. 2017 Nov 2;12(11):e0187410. doi: 10.1371/journal.pone.0187410 (PMC5667756; doi:10.1371/journal.pone.0187410)
Supplement: S2 Table — Numeric values are expressed as n (%), mean ± standard deviation or median (interquartile range 25–75%). Cut-off values in continuous values were decided based on maximum Youden’s indexes. Alb: Albumin; BUN: blood urea nitrogen; CRP: C-reactive protein; Hb: Hemoglobin; Max: maximum; Min: minimum; Plt: Platelets; T-Bil: total bilirubin; WBC: white blood cells. (DOCX) [file pone.0187410.s003.docx]

|  | Alive  [n=550] | Dead  [n=47] | *P* value | c statistics | Cut-off  Value |
| --- | --- | --- | --- | --- | --- |
| Catecholamine Administration (n) | 85 (15.5) | 28 (59.6) | <0.0001 | 0.721 |  |
| Respiratory Support (n) | 72 (13.1) | 16 (34.0) | 0.0005 | 0.605 |  |
| Min Alb (g/dL) | 2.70 ± 0.64 | 2.26 ± 0.69 | <0.0001 | 0.669 | 2.4 |
| Min Hb (g/dL) | 10.6 ± 2.4 | 9.1 ± 2.2 | <0.0001 | 0.678 | 9.4 |
| Min Plt (×10^4^/μL) | 16.2 [12.9-20.1] | 11.1 [6.8-14.1] | <0.0001 | 0.728 | 14.2 |
| Max WBC (×10^2^/μL) | 80.0 [65.0-104.0] | 100.0 [76.0-151.0] | 0.0027 | 0.632 | 107.0 |
| Max T-Bil (mg/dL) | 0.95 [0.71-1.42] | 1.45 [0.88-2.19] | <0.0001 | 0.675 | 1.24 |
| Max BUN (mg/dL) | 37 [27-51] | 79 [49-107] | <0.0001 | 0.799 | 61 |
| Max Creatinine (mg/dL) | 1.30 [0.98-2.00] | 2.34 [1.40-3.40] | <0.0001 | 0.730 | 2.17 |
| Min Sodium (mEq/L) | 137 [134-139] | 134 [131-137] | 0.0029 | 0.630 | 136 |
| Max CRP (mg/dL) | 2.33 [0.63-6.31] | 7.26 [2.77-14.77] | <0.0001 | 0.708 | 2.71 |
