# Supplementary material for: Novel risk stratification with time course assessment of in-hospital mortality in patients with acute heart failure
Source: PLoS One. 2017 Nov 2;12(11):e0187410. doi: 10.1371/journal.pone.0187410 (PMC5667756; doi:10.1371/journal.pone.0187410)
Supplement: S3 Table — AF: Atrial Fibrillation; CI: Confidence Interval; DM: Diabetes Mellitus; GWTG—HF: Get With The Guidelines—Heart Failure; LVEF: Left Ventricular Ejection Fraction; SR: Sinus Rhythm. (DOCX) [file pone.0187410.s004.docx]

|  |  | GWTG–HF risk score |  | In-Hospital risk score |  |
| --- | --- | --- | --- | --- | --- |
|  | (n) | C Statistics [95%CI] |  | C Statistics [95%CI] | *P* value |
| Overall | 1035 | 0.789 [0.737-0.842] |  | 0.894 [0.860-0.928] | <0.001 |
| Male | 542 | 0.817 [0.753-0.882] |  | 0.918 [0.879-0.957] | 0.003 |
| Female | 493 | 0.761 [0.680-0.842] |  | 0.871 [0.816-0.926] | <0.001 |
| Age<80 years | 534 | 0.826 [0.730-0.923] |  | 0.905 [0.849-0.960] | 0.054 |
| Age≥80 years | 501 | 0.731 [0.660-0.802] |  | 0.870 [0.822-0.919] | <0.001 |
| DM | 314 | 0.779 [0.687-0.872] |  | 0.889 [0.829-0.950] | 0.010 |
| AF | 362 | 0.843 [0.776-0.909] |  | 0.948 [0.906-0.990] | 0.004 |
| SR | 578 | 0.755 [0.679-0.832] |  | 0.873 [0.826-0.920] | <0.001 |
| LVEF ≥ 40% | 563 | 0.765 [0.690-0.840] |  | 0.884 [0.836-0.931] | <0.001 |
| LVEF < 40% | 472 | 0.825 [0.758-0.892] |  | 0.913 [0.870-0.957] | 0.006 |
